# Gamma Knife Radiosurgery does not alter the copy number aberration profile in sporadic vestibular schwannoma

**DOI:** 10.1007/s11060-020-03631-4

**Published:** 2020-09-27

**Authors:** Aril Løge Håvik, Ove Bruland, Dhanushan Dhayalan, Morten Lund-Johansen, Per-Morten Knappskog

**Affiliations:** 1 Department of Clinical Science, University of Bergen, Bergen, Norway; 2 Center for Medical Genetics and Molecular Medicine, Haukeland University Hospital, Bergen, Norway; 3 Department of Clinical Medicine, University of Bergen, Bergen, Norway; 4 Department of Neurosurgery, Haukeland University Hospital, Bergen, Norway

**Keywords:** Vestibular schwannoma, Gamma Knife Radiosurgery, Whole genome microarray, Intratumor genetic heterogeneity, Neurosurgery, Genetics

## Abstract

**Introduction:**

Ionizing radiation is a known etiologic factor in tumorigenesis and its role in inducing malignancy in the treatment of vestibular schwannoma has been debated. The purpose of this study was to identify a copy number aberration (CNA) profile or specific CNAs associated with radiation exposure which could either implicate an increased risk of malignancy or elucidate a mechanism of treatment resistance.

**Methods:**

55 sporadic VS, including 18 treated with Gamma Knife Radiosurgery (GKRS), were subjected to DNA whole-genome microarray and/or whole-exome sequencing. CNAs were called and statistical tests were performed to identify any association with radiation exposure. Hierarchical clustering was used to identify CNA profiles associated with radiation exposure.

**Results:**

A median of 7 (0–58) CNAs were identified across the 55 VS. Chromosome 22 aberration was the only recurrent event. A median aberrant cell fraction of 0.59 (0.25–0.94) was observed, indicating several genetic clones in VS. No CNA or CNA profile was associated with GKRS.

**Conclusion:**

GKRS is not associated with an increase in CNAs or alteration of the CNA profile in VS, lending support to its low risk. This also implies that there is no major issue with GKRS treatment failure being due to CNAs. In agreement with previous studies, chromosome 22 aberration is the only recurrent CNA. VS consist of several genetic clones, addressing the need for further studies on the composition of cells in this tumor.

## Introduction

Vestibular schwannoma (VS) is a benign intracranial neoplasm originating from the Schwann cells surrounding the vestibular portion of the 8th cranial nerve. VS makes up 8% of intracranial tumors, with an annual incidence rate ranging from 10 to 22 per million [1, 2]. Although patients have a normal life expectancy, they experience significantly reduced quality of life attributable to dizziness, headache, hearing loss, facial nerve palsy and tinnitus [3]. Inactivation of the *NF2* tumor suppressor gene is considered an initiating event in VS tumorigenesis, but it is likely that other factors also contribute [4, 5]. During recent years, several novel genetic events have been linked to the disease [6–8].

Gamma Knife Radiosurgery (GKRS) is a type of ionizing radiation therapy commonly used to treat VS. There are controversies regarding whether ionizing radiation might induce malignant degeneration or second neoplasms [9, 10]. The risk for inducing neoplasms following ionizing radiation demonstrates a linear dose-response relationship, thus making it theoretically feasible for GKRS inducing neoplasms in the normal tissue surrounding VS [11].

The aim of this study was to analyze the genome of 55 sporadic VSs (sVS) to assess whether GKRS induce copy number aberrations (CNA). To understand the mechanism, we wanted to evaluate whether there are any genetic aberrations associated with GKRS treatment resistance. Previous studies on structural changes in the VS genome have identified chromosome 22q loss as the only recurring event, present in 25–83% of VSs [12]. However, previous studies have used techniques with lower resolution. Hence, our secondary aim was to characterize CNAs at a more detailed level as well as using this data to evaluate intratumor heterogeneity.

## Materials and methods

### Patient samples

VS tissue and matched blood sample was collected from 55 patients without a history of NF2, who underwent first-time suboccipital resection of unilateral VS at the Department of Neurosurgery, Haukeland University Hospital, from August 2003 to May 2017. Eighteen patients had been previously treated with GKRS for the same VS. Written informed consent was received from all patients before tissue harvesting and the study was approved by the Regional Ethical Committee for medical research in Western Norway (2013/374). Tumor samples were harvested from the subcapsular part and snap frozen and stored in liquid nitrogen in the Bergen Neurosurgical Tissue Bank at Haukeland University Hospital. All samples underwent routine histology. Volumetric tumor measurements were performed on BrainLab Elements if preoperative MRI scans were available (Version 2.4.0, BrainLab AG, Munich, Germany).

### DNA extraction

DNA was extracted by disrupting the tumor tissue with the TissueLyser (Qiagen, Hilden, Germany) followed by protease treatment. DNA was then extracted using the QIAamp DNA Mini Kit (Qiagen). The DNA quality and quantity were evaluated with 1% SeaKem gel electrophoresis and NanoDrop (Thermo Fisher Scientific), respectively.

### Whole-genome DNA microarray

The CytoScan HD microarray (Affymetrix, UK) was used to detect chromosomal aberrations according to the manufacturer´s recommendations. CNAs were called using three different software: (1) chromosome analysis suite v3.2 (ChAS, Affymetrix, UK), (2) Rawcopy [13] and (3) Nexus Copy Number (BioDiscovery, El Segundo, CA, USA). All data were mapped to the hg 19 reference genome build. We applied the following filtering criteria for including the called CNAs in downstream analysis: (1) marker count ≥ 90 for gains; (2) marker count ≥ 30 for losses; (3) visual confirmation for mosaic variants; (4) segment size ≥ 1 Mbp for copy number neutral runs of homozygosity (CNN-ROH). Recurrent CNN-ROHs were further inspected for harboring small variants in whole-exome sequencing (WES) data using IGV[14]. BEDTools was used to produce a per sample union CNA call set, merge fragmented calls and to identify common regions harboring CNA across the cohort [15]. Candidate CNAs were manually inspected in IGV and filtered based on the following criteria: (1) variant not present in databases of copy number variants (CNV) in normal healthy controls (Affymetrix reference database with n = 2691, Database of genomic variants as per May 2016 [16]); (2) variant containing NCBI reference sequence gene; (3) variant present in 3 or more samples. GISTIC [17] was used to identify statistically significant aberrated regions across the cohort.

For estimating aberrant cell fraction and allele specific copy number profiles in the tumors, the Allele-Specific Copy number Analysis of Tumors 2.5.2 (ASCAT) software was used [18]. Per sample log ratio (LR) and B-allele frequency (BAF) values from the 27 tumors analyzed with Rawcopy was used for input. ASCAT was run with default parameters except from gamma which was set to 0.45 in compliance with the estimated compression factor in the Affymetrix CytoScan HD microarray.

### Whole-exome sequencing (WES)

WES data were available from a previous study on 46 sVSs, including 18 samples also analyzed with microarray [7]. The Sequenza software version 2.1.2 was used for estimating aberrant cell fraction and calling allele-specific copy number profiles from the BAM files [19]. The 18 samples analyzed with both microarray and WES were used as training data to set the following parameters for running Sequenza: gamma = 100, kmin = 30 and median normalization method. Aberrant cell fraction estimates below 0.20 were not included as the software was not trained to estimate at this level.

### Statistical analyses

Statistical analyses, including descriptive statistics, contingency table statistics, Mann-Whitney U test and linear correlation, were done using Nexus Copy Number and/or R [20]. Clustering of the sample set based on CNA profiles was done with Rawcopy using the hclust R package as well as with the built-in complete linkage hierarchical clustering algorithm in Nexus Copy Number.

## Results

### Patient characteristics

55 patients presenting with sVS were included (Table 1). Mean age at the time of surgery was 53.3 years ranging from 18 to 80 years. Mean preoperative tumor volume was 8.4 cm^3^ ranging from 0.37 to 26.78 cm^3^. 18 patients underwent Gamma Knife Radiosurgery (GKRS) prior to surgical removal of the primary tumor. Mean time between GKRS and surgery was 1429 days ranging from 280 to 3478 days, and the margin dose in all cases was 12 Gy. All but three GKRS treated patients needed surgical removal because of post-treatment growth; VS14 experienced dizziness, VS16 acquired an intratumoral cyst and VS26 developed trigeminal neuralgia. Five patients had cystic tumors.

**Table 1:** Patient demographics. Patient demographics of 55 vestibular schwannomas

ID	GKRS^1^	Age	Volume^2^	Sex	Microarray^3^	WES^4^
VS1	na	58	4.25	F	X	X
VS2	na	61	NA	M	X	X
VS3	na	68	16.34	M	X	X
VS4	na	67	3.17	F	X	X
VS5	na	58	12.82	M	X	X
VS6	na	57	NA	F	X	X
VS7	na	62	6.46	F	X	X
VS8	na	54	3.34	F	X	X
VS9	na	75	17.71	F	X	X
VS10	699	50	NA	F	X	X
VS11	1028	61	NA	M	X	X
VS12	3478	58	NA	M	X	X
VS13	1084	66	NA	M	X	X
VS14	2170	28	1.20	F	X	X
VS15	1079	64	NA	M	X	X
VS16	574	66	11.46	M	X	X
VS17	560	53	1.46	F	X	X
VS18	2371	69	1.67	F	X	X
VS19	1499	61	0.12	M	X	
VS20	280	44	0.62	M	X	
VS21	1476	66	3.50	M	X	
VS22	2968	80	0.41	M	X	
VS23	1987	60	0.47	M	X	
VS24	720	72	1.03	F	X	
VS25	811	68	0.14	M	X	
VS26	1646	61	4.56	F	X	
VS27	1288	61	2.52	M	X	
VS29	na	64	11.39	F		X
VS30	na	39	11.99	F		X
VS31	na	40	8.77	F		X
VS33	na	59	7.11	M		X
VS34	na	33	6.89	M		X
VS35	na	30	15.05	M		X
VS36	na	45	5.41	F		X
VS37	na	48	4.29	M		X
VS38	na	18	16.61	F		X
VS39	na	58	9.70	F		X
VS40	na	42	9.39	M		X
VS41	na	25	12.18	F		X
VS42	na	45	18.42	F		X
VS43	na	36	18.01	F		X
VS44	na	58	7.87	F		X
VS45	na	60	26.77	F		X
VS46	na	33	11.68	M		X
VS48	na	42	5.46	M		X
VS49	na	54	18.21	M		X
VS50	na	63	3.20	M		X
VS51	na	47	NA	M		X
VS52	na	55	6.80	M		X
VS53	na	66	8.57	M		X
VS54	na	37	12.94	F		X
VS55	na	26	10.40	F		X
VS56	na	57	10.66	M		X
VS57	na	63	6.73	M		X
VS58	na	42	5.01	F		X

^1^Time in days between Gamma Knife Radiosurgery and microsurgery

^2^Tumor volume in cm^3^

^3^Samples with DNA microarray data marked with X

^4^Samples with WES data marked with X

### Chromosome 22 aberration is the only recurrent copy number aberration in sVS

**Fig. 1:**
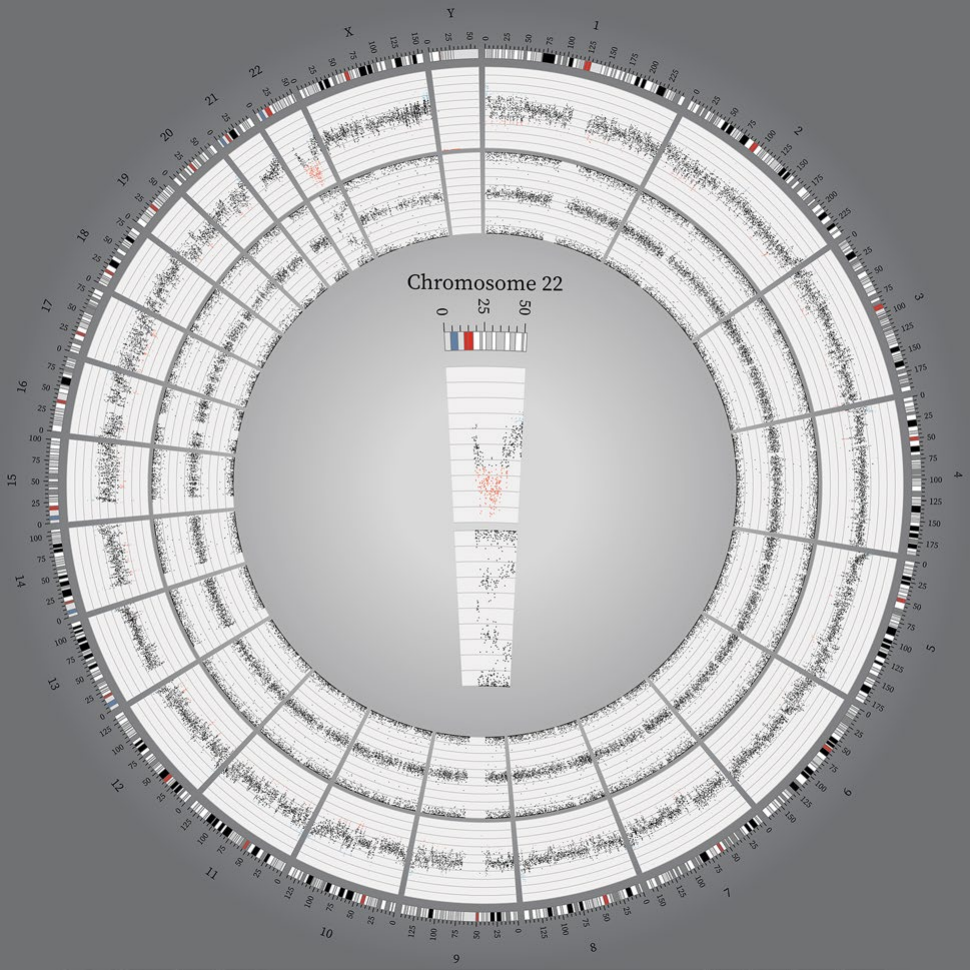
Karyogram for sample VS10. Circos plot of copy number and single nucleotide polymorphism probe data for sample VS10, created using the Circos software [37]. The tracks from outside inwards: chromosome numbers, chromosomal position in Mb, copy number and allele patterns. Copy number gains and losses are highlighted in blue and red, respectively. Most chromosomes show a continuous disomic copy number profile with a normal three band allele pattern (allele configurations AA, AB and BB). On chromosome 22, highlighted in the middle, we see an allelic loss (allele configuration A0 and B0) in the region of *NF2* followed by a CNN-ROH (allele configuration AA and BB). However, the aberrations are only present in 63% of the cells giving rise to the split in the middle line of the allele pattern

Using the union call set from filtered ChAS and Rawcopy segments, a median of 7 (0–58) CNAs per sample was identified. Figure 1 illustrates the karyogram of a representative sVS. A median of 0.17% of the sVS autosome was affected by CNA. 38 genomic loci were found to harbor a CNA in three or more samples. However, all but the chromosome 22 loss were common variants (CNV) present in healthy controls. A median of 3 (0–134) CNN-ROHs were seen in the tumors. None of the recurrent regions across the samples harbored any point mutations or indels. ASCAT was then used to infer aberrant cell fraction and absolute allele specific copy number. The number of CNAs identified by the different approaches were highly correlated (r = 0.831, p < 0.001). ASCAT identified a median of 21 (2–219) autosomal CNAs with a median gain-to-loss ratio of 1.25 (0.25–6.50). The only recurrent CNA retained after filtering was chromosome 22 loss or CNN-ROH. GISTIC analyses on segmented data from Rawcopy and Nexus Copy Number identified chromosome 22 loss as a significantly recurrent event (Q-bound = 1.36 × 10^− 9^, G-score = 15.72). Other events identified were either CNVs or non-coding DNA. Neither tumor volume, volumetric growth nor age was significantly associated with chromosome 22 status, number of CNAs or aberrant cell fraction. Aggregating the results from ASCAT and Sequenza, 25 out of 55 (45%) tumors harbored a chromosomal aberration at chromosome 22 including seven tumors with CNN-ROH, 17 tumors with loss and one tumor with a loss followed by a CNN-ROH (Table 2). Most aberrations encompassed all the analyzed probes on the chromosome suggesting a total loss of the chromosome. The chromosome 22 aberrated group was comparable to the entire cohort with regards to sex distribution, GKRS exposure, age, tumor volume and time elapsed from GKRS to surgery. Combining the structural variants identified in this study with whole-exome sequencing and multiplex ligation-dependent amplification (MLPA) data from our previous study, 41 out of 55 (75%) harbored at least one *NF2* mutation [7]. When only including the samples that were analyzed with whole-exome sequencing, 38 out of 46 (83%) harbored at least one mutation including 13 samples with one hit and 25 samples with 2 hits.

**Table 2:** Chromosome 22 aberrations

ID	Aberration	Aberrant cell fraction
VS1	22q11.1q13.33(16052530–51244019) × 1	0.86
VS8	22q11.1q13.33(16052530–51244019) × 1	0.46
VS9	22q11.1q13.33(17922735–51244019) hmz	0.46
VS10	22q11.1q13.33(19639383–37988033) × 1 22q11.1q13.1(37988034–51244019) hmz	0.63
VS13	22q11.1q13.33(16052530–51244019) × 1	0.61
VS16	22q11.1q13.33(16052530–51244019) × 1	0.46
VS17	22q11.1q13.33(16052530–51244019) × 1	0.29
VS20	22q11.1q13.33(16052530–51244019) × 1	0.26
VS22	22q11.1q13.33(16052530–51244019) × 1	0.45
VS27	22q11.21q13.33(18581773–51244019) hmz	0.25
VS33	22q11.1q13.33(16157603–51220938) × 1	0.63
VS34	22q11.1q13.33(16157940–51237063) × 1	0.59
VS37	22q11.1q13.33(16157827–51220938) hmz	0.36
VS38	22q11.23q12.3(24167473–33156768) hmz	NA
VS42	22q11.1q13.33(16157762–51220938) × 1	0.27
VS43	22q11.1q13.33(20761063–51220938) × 1	0.94
VS45	22q11.1q13.33(16157623–51237063) × 1	0.79
VS46	22q11.22q13.33(22313733–51237063) hmz	NA
VS50	22q11.1q13.33(16157622–51237063) × 1	0.77
VS51	22q11.1q13.33(16157603–51219006) × 1	0.66
VS53	22q11.1q13.33(26688838–51237063) × 1	0.8
VS54	22q11.1q13.33(16157603–51237063) × 1	NA
VS55	22q11.1q13.33(16157771–51220938) hmz	NA
VS57	22q11.23q13.33(23523234–51220938) hmz	0.4
VS58	22q11.1q13.33(16269779–51216564) × 1	0.84

Chromosome 22 aberrations identified in VS. The naming of the aberrations starts with chromosome number followed by band, location in bp and type of aberration (x1 for hemizygous loss and hmz for copy number neutral run of homozygosity). The last column gives the fraction of cells harboring the aberration

### Mosaic chromosome 22 loss reveals intratumor genetic heterogeneity in sVS

**Fig. 2:**
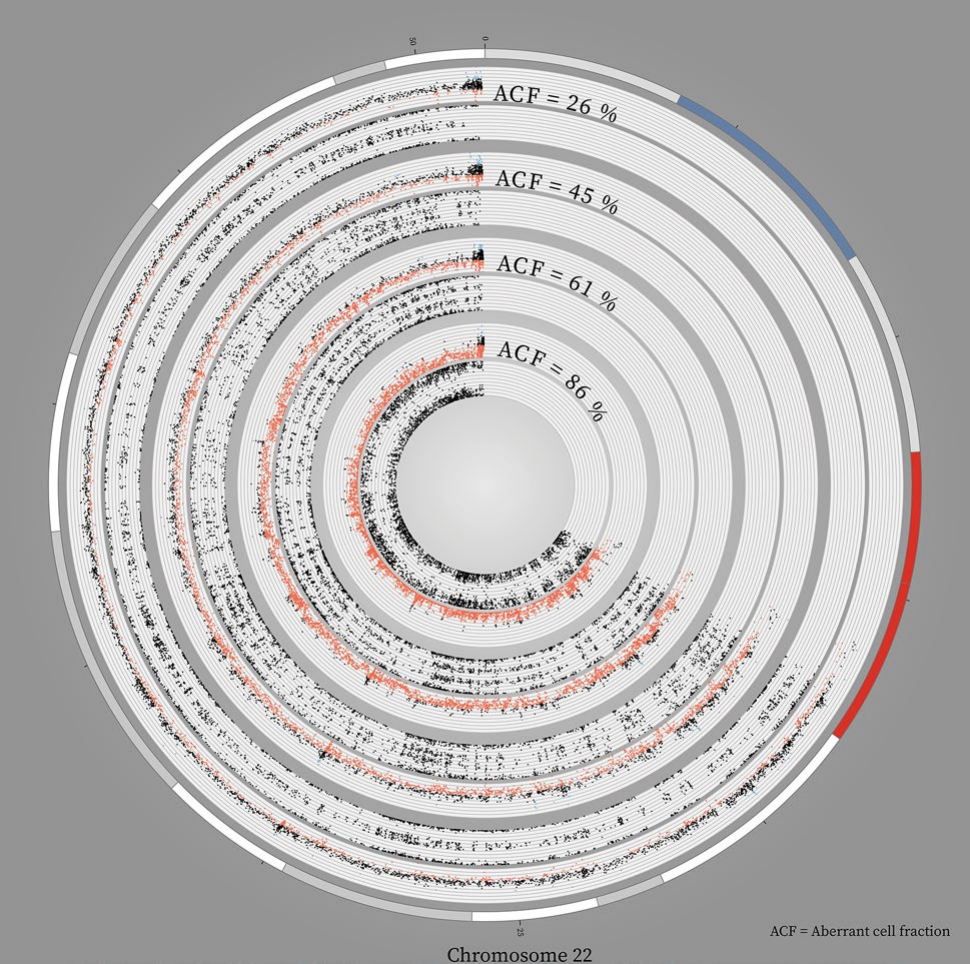
Vestibular schwannoma consist of more than one major genetic clone. Circos plot of copy number and single nucleotide polymorphism probes in chromosome 22 for four vestibular schwannomas with increasing aberrant cell fraction. The tracks from outside inwards: chromosomal position in Mb on chromosome 22, copy number and allele patterns respectively for four vestibular schwannomas with increasing aberrant cell fraction. All samples demonstrate hemizygous loss of chromosome 22. The outermost sample shows a minor drop in copy number and a barely visible split in the middle line in the allele pattern because only 26% of the cells are aberrated. Moving inwards, the copy number drops and the split in the allele pattern increases, demonstrating an increase in aberrant cell fraction

Among the chromosome 22 aberrated tumors, a median aberrant cell fraction of 0.59 (0.25–0.94) was observed (Table 2). Aberrant cell fraction did not correlate with tumor growth. The level of mosaicism is demonstrated in the splitting of the BAF signal (Fig. 2). A Chi-square test demonstrated that tumors with aberrated chromosome 22 were significantly more likely to be estimated as heterogeneous (*χ*^2^ *= *22.212,* Fisher’s p = *0.000). Among the four tumors with diploid chromosome 22 and estimated aberrant cell fraction below 1, one tumor had several CNN-ROHs, one tumor harbored another large CNA [del [21] (q11.2q22.3)], whereas the two other tumors were estimated to have an aberrant cell fraction between 0.95 and 1. It seems likely that ASCAT and Sequenza are dependent on a large CNA to estimate aberrant cell fraction and hence only estimates from chromosome 22 aberrant tumors were included for reporting (Table 2). In four chromosome 22 aberrated tumors, an aberrant cell fraction could not be estimated, and all these tumors had WES data only.

Among the 46 tumors analyzed with WES, a total of 45 small nucleotide variants and indels were found with a median variant allele frequency of 24% [7]. We found a positive correlation between the estimated aberrant cell fraction from the chromosome 22 aberrated tumors and variant allele frequency (adj R^2^ = 0.43, p = 0.006).

### GKRS does not alter the copy number profile of sVS

**Fig. 3:**
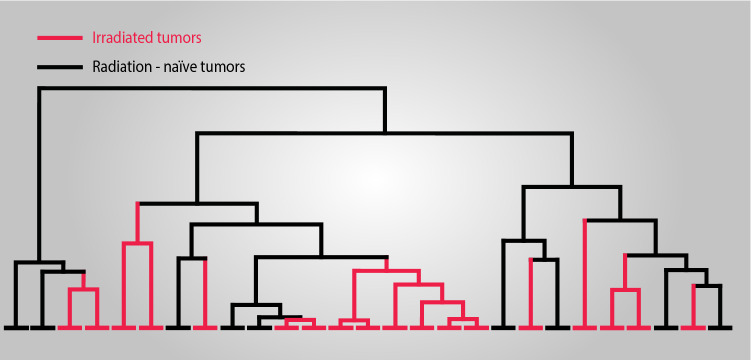
GKRS treatment does not affect the genomic CNA profile of vestibular schwannoma. Dendrogram of hierarchical clustering of the autosomal CNA patterns of vestibular schwannomas. Irradiated and radiation-naïve tumors depicted as red and black terminal vertical lines respectively. The clusters are not associated with previous radiation exposure

A Chi-square test of independence demonstrated no difference in the frequency of chromosome 22 aberration in irradiated (39%) and radiation-naïve (49%) tumors (*χ*^2^ *= *0.155, *Fisher’s p =* 0.572). We found no differences in aberrant cell fraction, number of CNAs, type of CNA or the portion of the genome covered by CNAs between the irradiated and radiation-naïve tumors. The clustering algorithms applied demonstrated that clusters identified did not rely on previous radiation exposure (Fig. 3). Using the Nexus Copy Number built-in comparison analysis, we did not identify any CNA or gene associated with radiation exposure. We sought specifically for CNAs affecting genes coding for enzymes annotated to function in DNA repair pathways. Using the union call set, four tumors (two irradiated and two radiation-naïve) harbored CNAs affecting DNA repair genes. No difference between the groups was seen (x^2^ = 0.04, p = 0.58). The results were similar for the ASCAT call set.

## Discussion

GKRS has become increasingly popular in treating VS over the past decades [21]. Several case reports have questioned its safety regarding malignant degeneration of benign tumors and inducing new neoplasms. For a review, see [9]. A dominating theory explaining the relationship between ionizing radiation dose and harmful effects is called the “linear no-threshold model” [22]. Although disputed, this theory explains that there is no safe limit, and that even a small amount of radiation might damage the DNA and initiate tumorigenesis. Standard treatment protocol with GKRS delivers 12 Gy to the periphery with a sharp decrease in the amount of energy delivered to the surrounding tissue. In the focus of the radiation, the dosage will be enough to initiate necrosis. In the periphery, we hypothesize that the dosage will harm the DNA and lead to one of the following: (1) detrimental DNA damage leading to apoptosis, (2) DNA mutations with tumorigenic potential, (3) DNA mutations with other or no effect at all. Although it seems theoretically feasible for GKRS to increase the risk of malignancy, epidemiologic studies do not support this [10, 23].

Previous studies have reported different genetic findings associated with radiation in VS. Lee et al. analyzed 30 sVS, including 4 irradiated tumors, utilizing microsatellite analysis to find that chromosome 22 aberration was more common in the radiation-naïve tumors [24]. Warren et al. found, using comparative genomic hybridization, that among 10 neurofibromatosis type 2 patients, radiation was associated with chromosomal aberrations [25]. In a recent study, Aaron et al. used WES on 12 VS, including two irradiated, to conclude that irradiated VS have increased copy number events and mutational burden [26]. One irradiated tumor harbored 184 mutations whereas the average across the cohort was 18.5. However, in our previous study utilizing WES on 46 VS, including 8 irradiated tumors, we also demonstrated one outlier, but this was radiation-naïve [7]. Taken together, this weakens the association between radiation exposure and hypermutated tumors.

This is the largest study investigating the effect of GKRS on the sVS genome. Using hierarchical clustering of the genome-wide CNA profiles, we did not identify any clustering based on radiation exposure. Neither did we find any gene or genomic loci that correlated with radiation exposure. The discrepancy with previous studies on the subject might be due to larger sample size and the method used. In a recent paper, 18 radiation-induced meningiomas were analyzed for tumor-specific CNAs [27]. A mean total of 22% of the exome was affected by CNA. This is in stark contrast with our irradiated tumors exhibiting a median of 0.14% of the autosome covered by CNA. The meningioma patients had received cranial radiotherapy for diseases like medulloblastoma and central nervous system lymphoma, a therapy that delivers higher radiation doses to healthy tissue compared to GKRS. This comparison lends support to the fact that GKRS does not induce collateral damage to the extent seen after conventional radiotherapy. We expect that GKRS causes mutations in the normal tissue surrounding VS. However, the mutations induced need to provide a selective growth advantage to the affected cell initiating a clonal expansion for it to be detected using bulk DNA analysis and even for it to be clinically relevant. It is feasible that a growth advantage might be obtained, but that it is very rare in agreement with epidemiologic studies and our study.

Between 5 and 10% of sVSs do not respond to GKRS treatment. The GKRS response might depend on both treatment and tumor factors. Studying the tumor factors might elucidate the mechanism of radioresistance as well as identify biomarkers. Archibald et al. found a higher expression of the immune-related protein B7-H1 among irradiated sVS, but no difference at RNA level [28]. This might be a consequence of the radiation induced inflammation and hence not connected to the cause of the radioresistance. Through the use of genome-wide association studies, gene expression and DNA sequencing, several biomarkers of radiotherapy treatment response have been found in neoplasms [29–32]. It has been postulated that enhanced DNA repair mechanisms lead to radiotherapy treatment failure. Hence, we sought to evaluate whether the radioresistant VSs harbored CNAs in DNA repair genes. Although we identified some impaired DNA repair genes, they were distributed equally among the irradiated and radiation-naïve tumors. We did not find any other gene or genomic loci associated with radioresistance. One pitfall of our study is that we do not have positive controls for tumors that respond to GKRS treatment as these are not surgically removed. However, as GKRS treatment is effective in 90–95% of sVSs, we believe that the radiation-naïve tumors included in this study serve as a viable surrogate for GKRS treatment responders. Also, to detect any genetic aberrations caused by ionizing radiation, a longitudinal study design using paired samples of radiation-naïve and irradiated tumors would be the most sensitive. However, we believe our study design would be able to detect any large effects ionizing radiation.

Among the chromosome 22 aberrated tumors, a median of 59% of the cells harbored the CNA, suggesting that VS consists of more than one major clone. This is in accordance with our previous study on small mutations, where we reported a median *NF2* variant allele frequency of 24% [7]. Considering the bias of ASCAT only calling heterogeneity in tumors with large aberration and the variant allele frequencies reported in our WES study, it seems likely that most or all VSs consists of more than one major genetic clone. A recent study by Lewis et al. found that tumor-associated macrophages constituted 50–60% of the cells in eight growing VSs [33]. Hence, infiltrating macrophages might constitute the clone coexisting with the neoplastic cells. Further on, Lewis et al. found that macrophages accounted for the proliferating cells in VS. However, we did not find any association between aberrant cell fraction, which might correlate inversely with the macrophage fraction, and tumor growth. The finding of intratumor genetic heterogeneity has significant implications for molecular studies on VS. We saw from our data that the lower the aberrant cell fraction, the higher the number of CNAs called. This implies a problem with the software and theoretic framework underlying calling of aberrations. The possibly large fraction of infiltrating macrophages would also preclude other molecular studies using bulk analyzing, like transcriptome and proteome studies. A possible way to bypass this could be to apply single-cell analysis to provide a better understanding VS molecular biology.

Carlson et al. recently profiled structural variants in sVS using whole-genome sequencing to find biallelic inactivation of the *NF2* gene in all 22 sVSs analyzed [8]. Previous studies have demonstrated *NF2* variants in 15–84% of the analyzed tumors [34]. Combining our data from whole-genome microarray, WES and MLPA, we found at least one variant in *NF2* in 83% of the tumors. This discrepancy might reflect differences in the detection limit of the methods used or variations in the study population. Carlson et al. also found that VSs with severe phenotype tended to harbor large structural variants outside chromosome 22. However, we did not find any association with specific CNAs and previous radiation exposure, tumor size or age. In agreement with Carlson et al. we did not find any recurrent focal alterations and it seems unlikely that this kind of genetic event plays a significant role in VS tumorigenesis [8]. Previous studies have identified recurrent non-chromosome 22 regions affected by CNA, like 9q34, 17q, 19, 16q and 9p21 [25, 35]. We found a total of 38 genomic loci affected in three or more tumors. However, all but chromosome 22 aberration were normal variants present in healthy subjects.

In our previous study, we analyzed a total of 46 VS, including 8 irradiated VS, with WES and MLPA to conclude that radiation exposure or radiosensitivity is not associated with increased mutational burden or specific small mutations [7]. Ionizing radiation is known to induce DNA double-strand breakage resulting in CNA [36]. To address this issue, we analyzed an extended set of irradiated samples with whole-genome DNA microarray to conclude that neither specific CNAs, nor the genomic CNA profile play a role either. The methods used in these studies are not capable of detecting structural variants not affecting gene dosage or heterozygosity (e.g. inversions and translocations). Hence, future studies should address this as well as epigenetic mechanisms to elucidate the molecular consequences of ionizing radiation in VS as well as markers of radioresistance.

## Conclusions

We did not find any CNA or genomic CNA profile associated with radiation exposure in VS. This finding lends support to the low risk of GKRS. We demonstrated that VS exhibit intratumor heterogeneity and further studies are warranted to elucidate whether it is different tumor clones or normal cell infiltration. In our study, the only recurrent CNA in VS is hemizygous loss or copy number neutral loss of heterozygosity on chromosome 22.
